# Effect of Acupuncture in Mild Cognitive Impairment and Alzheimer Disease: A Functional MRI Study

**DOI:** 10.1371/journal.pone.0042730

**Published:** 2012-08-20

**Authors:** Zhiqun Wang, Binbin Nie, Donghong Li, Zhilian Zhao, Ying Han, Haiqing Song, Jianyang Xu, Baoci Shan, Jie Lu, Kuncheng Li

**Affiliations:** 1 Department of Radiology, Xuanwu Hospital of Capital Medical University, Beijing, China; 2 Institute of High Energy Physics, Chinese Academy of Sciences, Beijing, China; 3 General Hospital of Chinese People's Armed Police Forces, Beijing China; 4 Department of Neurology, Xuanwu Hospital of Capital Medical University, Beijing, China; 5 Key Laboratory for Neurodegenerative Diseases, Ministry of Education, Beijing, China; Institute of Psychology, Chinese Academy of Sciences, China

## Abstract

We aim to clarify the mechanisms of acupuncture in treating mild cognitive impairment (MCI) and Alzheimer disease (AD) by using functional magnetic resonance imaging (fMRI). Thirty-six right-handed subjects (8 MCI patients, 14 AD patients, and 14 healthy elders) participated in this study. Clinical and neuropsychological examinations were performed on all the subjects. MRI data acquisition was performed on a SIEMENS verio 3-Tesla scanner. The fMRI study used a single block experimental design. We first acquired the baseline resting state data in the initial 3 minutes; we then acquired the fMRI data during the procession of acupuncture stimulation on the acupoints of Tai chong and Hegu for the following 3 minutes. Last, we acquired fMRI data for another 10 minutes after the needle was withdrawn. The preprocessing and data analysis were performed using the statistical parametric mapping (SPM8) software. Then the two-sample t-tests were performed between each two groups of different states. We found that during the resting state, brain activities in AD and MCI patients were different from those of control subjects. During the acupuncture and the second resting state after acupuncture, when comparing to resting state, there are several regions showing increased or decreased activities in MCI, AD subjects compared to normal subjects. Most of the regions were involved in the temporal lobe and the frontal lobe, which were closely related to the memory and cognition. In conclusion, we investigated the effect of acupuncture in AD and MCI patients by combing fMRI and traditional acupuncture. Our fMRI study confirmed that acupuncture at Tai chong (Liv3) and He gu (LI4) can activate certain cognitive-related regions in AD and MCI patients.

## Introduction

Alzheimer's disease (AD) is one of the most prevalent forms of dementia worldwide. The neuropathological changes of AD are characterized by amyloid-β plaques, neurofibrillary tangles and neuronal loss [1]. Mild cognitive impairment (MCI) is the most important at-risk state of AD. It has a high probability of degenerating into AD at a rate of 10–15% per year [2]. However, There are no effective therapy for AD and MCI. Acupuncture, a treatment of traditional Chinese medicine (TCM), remains promising as an investigational therapy to treat neurological diseases including chronic pain, drug addiction, stroke as well as dementia [3]–[5]. Despite its increasing usage of acupuncture, its underlying mechanisms are poorly understood.

Most recent findings hint that sensitive neuroimaging and network analysis played a special role for understanding the pathophysiological mechanism of MCI and AD. Several resting-state fMRI studies have investigated the neuronal integrity in the brain of the AD or MCI patients by different methods. By using regions of interest (ROI)-based functional connectivity approaches, the researchers found reduced functional integrity related to hippocampus [6]–[8], prefrontal regions [9] and posterior cingulate cortex (PCC) [10], [11] in AD or MCI patients. Using independent component analysis (ICA), Greicius and colleagues [12] showed AD-related reduction of spontaneous brain activity within a default-mode network (DMN) including the PCC and medial prefrontal cortex (MPFC). Sorg et al. [13] found the DMN regions and executive attention network had markedly reduced brain activity in the MCI patients.

Neuroimaging, in particular functional magnetic resonance imaging (fMRI), is a versatile tool that has been applied to investigate the mechanisms of acupuncture. Accumulating neuroimaging studies in humans have shown that acupuncture can modulate a widely distributed brain network [14]–[23], for example, Feng et al. [14] sought to investigate the functional correlations throughout the entire brain following acupuncture at acupoint ST36, they found that increased correlations for acupuncture were primarily related with the limbic/paralimbic and subcortical regions, whereas decreased correlations were mainly related with the sensory and frontal cortex. Zhong et al. [15] investigated modulatory effects of acupuncture at GB40 (Qiuxu) and KI3 (Taixi) on resting-state networks and found that acupuncture at different acupoints could exert different modulatory effects. Zhang et al [23] found that stimulating PC6 (Neiguan) can change the amplitude of the intrinsic cortical activity of the brain. They concluded that stimulating PC6 may be a candidate method for improving cognitive impairment due to the consistent effect of acupuncture within PCC.

Based on the above knowledge, we can speculate that acupuncture may have a great effect on patients such as AD and MCI through modulating special brain network or brain regional activity. However, most of the acupuncture studies have been performed on healthy subjects, to the best of our knowledge; only two fMRI studies have been published on acupuncture effect in patients with AD and MCI [24]–[25].One previous study found that the temporal lobe, some regions of parietal lobe and cerebellum could be activated by acupuncture in AD patients [24]. Another recent fMRI study on MCI patients found the enhanced correlations in the memory-related brain regions following acupuncture [25]. In order to better understanding of the pathophysiology of AD and MCI, we sought to investigate the effect of acupuncture on the brain functional activity throughout the entire brain in AD and MCI patients compared to normal controls. We first identified regions showing abnormal brain activity in AD and MCI patients comparing to controls during the resting state. After that, we tested whether these regions could be modulated in AD and MCI patients in the procession of acupuncture. Finally, we explored whether there were any alterations or specific modulatory patterns after the acupuncture in AD and MCI patients by comparing the poststimulus resting state with the resting state.

## Materials and Methods

### Subjects

Thirty-six right-handed subjects participated in this study after giving written informed consent, including 14 patients with AD, 8 patients with MCI and 14 healthy controls. This study was approved by the Medical Research Ethics Committee of Xuanwu Hospital. The AD and MCI subjects were recruited from patients who had consulted the memory clinic at Xuanwu Hospital for memory complaints. The healthy elderly controls were recruited from the local community.

All AD patients underwent a complete physical and neurological examination, standard laboratory tests and an extensive battery of neuropsychological assessments. The diagnosis of AD fulfilled the Diagnostic and Statistical Manual of Mental Disorders 4th Edition criteria for dementia (American Psychiatric Association, 1994), and the National Institute of Neurological and Communicative Disorders and Stroke/Alzheimer Disease and Related Disorders Association (NINCDS-ADRDA) criteria for possible or probable AD (McKhann et al., 1984). The subjects were assessed with the Clinical Dementia Rating (CDR) score [26], CDR of 1 and 2 was assigned to the AD category.

Participants with MCI had memory impairment but did not meet the criteria for dementia. The criteria for identification and classification of subjects with MCI [27] was: a) impaired memory performance on a normalized objective verbal memory test; b) recent history of symptomatic worsening in memory; c) normal or near-normal performance on global cognitive tests (MMSE score>24), as well as on an activities of daily living scale; (d) global rating of 0.5 on the CDR Scale, with a score of at least 0.5 on the memory domain; e) absence of dementia.

Healthy controls met the following criteria: a) no neurological or psychiatric disorders such as stroke, depression and epilepsy; b) no neurological deficiencies such as visual or hearing loss; c) no abnormal findings such as infarction or focal lesion in conventional brain MR imaging; d) no cognitive complaints; e) MMSE score of 28 or higher; f) CDR score of 0.

Participants with contraindications for MRI such as pacemaker, cardiac defibrillator, implanted material with electric or magnetic system, vascular clips or mechanical heart valve, cochlear implant or claustrophobia were excluded. In addition, patients with a history of stroke, psychiatric diseases, drug abuse, severe hypertension, systematic diseases and intellectual disability were excluded.

### Data acquisition

MRI data acquisition was performed on a SIEMENS verio 3-Tesla scanner (Siemens, Erlangen, Germany). The subjects were instructed to hold still, keep eyes closed and think nothing in particular. fMRI was acquired axially using an echo-planar imaging (EPI) [repetition time (TR)/echo time (TE)/flip angle (FA)/field of view (FOV) = 2000 ms/40 ms/90°/24 cm, image matrix = 64×64, slice number = 33, thickness = 3 mm, gap = 1 mm, bandwidth = 2232 Hz/pixel]. In addition, 3D T_1_-weighted magnetization-prepared rapid gradient echo (MPRAGE) sagittal images were obtained (TR/TE/inversion time (TI)/FA = 1900 ms/2.2 ms/900 ms/9°, image matrix = 256×256, slice number = 176, thickness = 1 mm).

**Figure 1:**
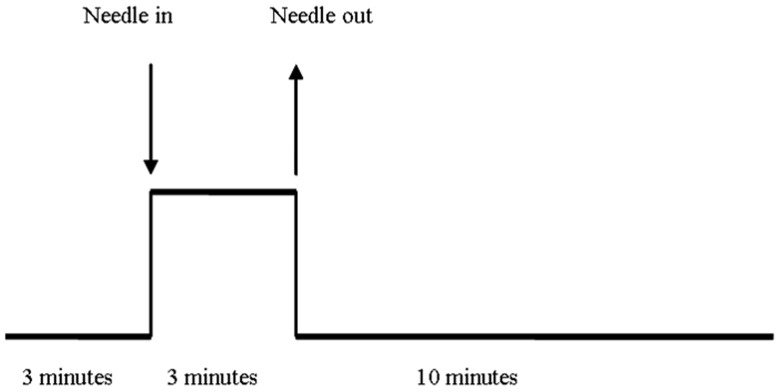
Experimental paradigm.

Our study used a single block experimental design. We first acquired the baseline resting state data in the initial 3 minutes; we then acquired the fMRI data during the procession of acupuncture stimulation for the following 3 minutes. A silver needle of 0.30 mm in diameter and 25 mm long was inserted and twirled at the four acupoints of the human body -Tai chong (Liv3) on the dorsum of the left and right foot; He gu (LI4) on the dorsum of the left and right hand. We acquired fMRI for another 10 minutes after the needle was withdrawn (Figure 1).

### Data analysis

fMRI post-processing was performed by a single experienced observer, unaware to whom the scans belonged. The preprocessing and data analysis were performed using the statistical parametric mapping (SPM8) software (Wellcome Department of Imaging Science; http://www.fil.ion.ucl.ac.uk/spm). The functional datasets of all patients and healthy controls were pre-processed using the following main steps. 1) Slice timing: the differences of slice acquisition times of each individual were corrected using slice timing. 2) Realign: the temporal processed volumes of each subject were realigned to the first volume to remove the head motion, and a mean image was created over the 317 realigned volumes. All participants had less than 3 mm of translation in x, y, or z axis and 1° of rotation in each axis. 3) Spatial normalization: the realigned volumes were spatially standardized into the MNI (Montreal Neurological Institute) space by normalizing with the EPI template via their corresponding mean image. Then, all the normalized images were resliced by 3.0×3.0×3.0 mm^3^ voxels. 4) Smooth: the normalized functional series were smoothed with a Gaussian kernel of 8 mm full width at half-maximum (FWHM).

The first level, for each smoothed individual image, was fixed effects analysis based on the general linear model with a box-car response function as the reference waveform convolved with the canonical hemodynamic response function. There are three experimental conditions comprising resting state (baseline), acupuncture stimulation and the second resting state after withdraw of the acupuncture needle. The contrasts of cerebral areas activation during these three conditions were created. The subject-specific contrast images were then used to perform the second level analysis based on the random effects. The two-sample t-tests were performed (1) between AD and healthy controls of baseline; (2) between MCI and healthy controls of baseline; (3) between acupuncture stimulation and baseline of AD group; (4) between acupuncture stimulation and baseline of MCI group; (5) between the resting state after withdraw of the acupuncture needle and baseline of AD group; (6) between the resting state after withdraw of the acupuncture needle and baseline of MCI group. Brain regions with significant BOLD changes in patients of all the six statistical analysis demonstrated above were yielded based on a voxel-level height threshold of p<0.001 (uncorrected) and a cluster-extent threshold of 5 voxels.

## Results

### Demography and neuropsychological test

Demographic characteristics and neuropsychological scores were shown in Table 1. There were no significant differences among the three groups in gender, age, and years of education, but the neuropsychological test such as Mini-Mental State Examination (MMSE) and Auditory verbal learning test (AVLT) scores were significantly different (*P*<0.01) among the three groups.

**Table 1:** Characteristics of the AD, MCI patients and Normal controls.

Characteristics	AD	MCI	NOR	*P* value
N (M/F)	14(4/10)	8(3/5)	14(6/8)	-
Age, years	66.92±8.91	66.37±10.96	66.07±5.78	0.96*
Education, years	10.07±3.38	10.62±3.54	11.00±4.52	0.82*
MMSE	15.92±4.32	25.37±1.30	28.00±1.41	<0.01*
AVLT(immediate)	11.35±3.95	14.13±3.52	26.86±5.24	<0.01*
AVLT(delayed)	2.64±1.59	4.37±1.59	11.07±2.76	<0.01*
AVLT(recognition)	3.35±1.55	7.38±3.11	12.71±2.09	<0.01*
CDR	1–2	0.5	0	-

MMSE, Mini-Mental State Examination; Plus-minus values are means ± S.D. AVLT, Auditory verbal learning test; immediate, immediate recall of learning verbal; delayed; delayed recall of learning verbal; recognition, recognition of learning verbal; CDR, clinical dementia rate.

* The *P* values were obtained by one-way analysis of variance tests.

### Regions showing increased or decreased activities in MCI, AD subjects comparing to normal subjects in resting state

**Figure 2:**
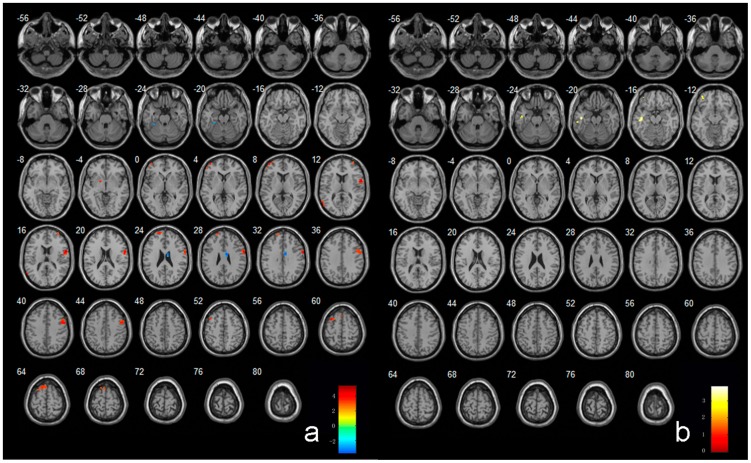
Regions showing abnormal activities in MCI subjects (a) and AD subjects (b) in resting state. Left in picture is left in the brain. The color scale represents t values.

When compared to normal subjects, increased activities in MCI patients were found in regions of the temporal lobe [left middle temporal gyrus(MTG) ], frontal lobe [left superior frontal gyrus(SFG), left middle frontal gyrus (MFG) and bilateral inferior frontal gyrus(IFG) ] and left lentiform nucleus; while decreased activities in MCI patients were found in regions of right cingulate gyrus and left fusiform gyrus(FG). In AD patients, left temporal lobe and left MFG showed decreased activities from that of normal subjects. The details of these regions see table 2 and Figure (2a, 2b).

**Table 2:** Regions showing increased or decreased activities in MCI, AD subjects comparing to normal subjects in resting state.

Regions	BA	Cluster	Coordinates (MNI)			T-score
		Size	x	y	z	
**MCI vs. NOR**						
Lt. Middle Temporal Gyrus ↑	39	16	−57	−67	13	3.94
Rt. Inferior Frontal Gyrus↑	44	127	60	5	16	5.46
Lt. Middle Frontal Gyrus ↑	6	8	−33	14	61	4.05
Lt. Middle Frontal Gyrus ↑	10	19	−39	59	7	3.78
Rt. Inferior Frontal Gyrus ↑	45	6	57	29	7	3.64
Lt. Inferior Frontal Gyrus↑	46	7	−48	47	4	3.37
Lt. Superior Frontal Gyrus↑	10	12	−18	62	25	3.30
Lt. Superior Frontal Gyrus↑	6	16	−15	23	64	3.15
Lt. Lentiform Nucleus↑	-	6	−15	2	−5	3.28
Rt. Cingulate Gyrus↓	-	16	12	−4	28	−3.19
Lt. Fusiform Gyrus↓	20	6	−30	−37	−23	−3.15
**AD vs. NOR**						
Lt. Temporal Lobe ↓	20	45	−42	−19	−17	−4.11
Lt. Middle Frontal Gyrus↓	11	12	−36	50	−14	−3.19
**AD vs. MCI**						
Lt. Middle Temporal Lobe↓	21	22	−48	−34	−2	−4.87
Lt. Middle Temporal Lobe↓	21	8	−54	5	−23	−3.55
Lt. Inferior Parietal lobule↓	40	54	−60	−49	43	−3.89
Lt. Middle Frontal Gyrus ↓	11	29	−30	35	−14	−3.54
Rt. Precentral gyrus ↓	6	8	63	−1	31	−3.34
Lt. Frontal Sub Gyral ↓	-	9	−12	20	−8	−3.24
Lt. Superior Frontal Gyrus↓	8	6	−33	20	58	−3.08

### Regions showing increased or decreased activities in MCI and AD subjects in the procession of acupuncture comparing to resting state

**Figure 3:**
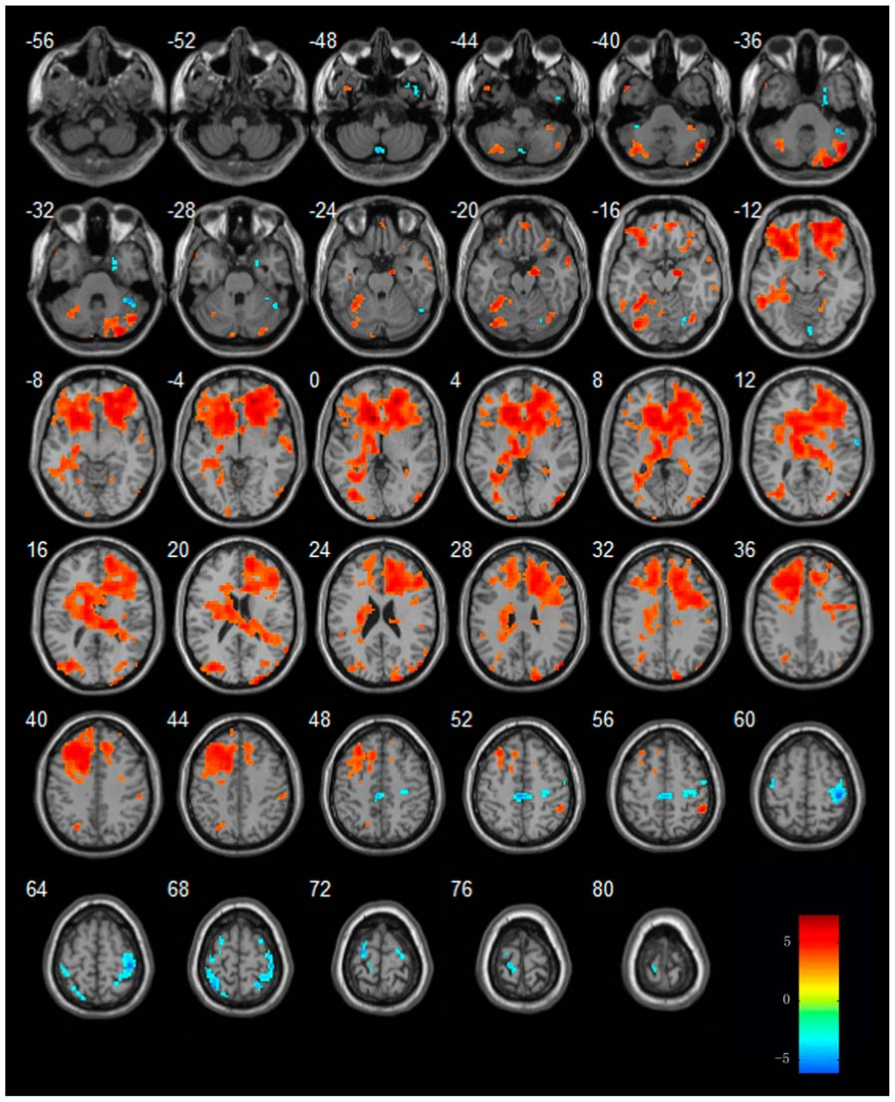
Regions showing increased or decreased activities in MCI subjects in the procession of acupuncture comparing to resting state. Left in picture is left in the brain. The color scale represents t values.

When compared to resting state, MCI patients showed increased activities in regions of bilateral cerebellum posterior lobe (CPL), temporal lobe [bilateral MTG, bilateral FG, right parahippocampus (PHG), left inferior temporal gyrus(ITG) ], frontal lobe, parietal lobe[bilateral inferior parietal lobule (IPL) and right postcentral gyrus(PoCG) ] and occipital lobe. Additionally, MCI patients showed decreased activities in regions of bilateral CPL, temporal lobe, frontal lobe [(bilateral SFG, right MFG, left precentral gyrus (PrCG)], parietal lobe [(right PoCG, left paracentral lobule (PCL), left superior parietal lobule (SPL)], right lingual gyrus and limbic regions. The details of these regions see table 3 and Figure 3

**Table 3:** Regions showing increased or decreased activities in MCI subjects in the procession of acupuncture comparing to resting state.

Regions	BA	Cluster	Coordinates (MNI)			T-score
		Size	x	y	z	
**MCI vs. NOR**↑						
Lt. Cerebellum Posterior lobe↑	-	12	−27	−49	−50	4.77
Lt. Cerebellum Posterior lobe↑	-	109	−36	−70	−41	4.45
Rt. Cerebellum Posterior lobe↑	-	231	51	−64	−38	6.81
Rt. Cerebellum Posterior lobe↑	-	11	36	−40	−44	3.78
Lt. Cerebellum Posterior lobe↑	-	12	−9	−91	−26	4.03
Lt. Cerebellum Posterior lobe↑	-	6	−3	−61	−17	3.46
Lt. Middle Temporal Gyrus↑	38	24	−48	11	−41	4.35
Lt. Fusiform Gyrus↑	19	92	−36	−76	−17	5.55
Lt. Inferior Temporal Gyrus↑	20	5	−39	−13	−26	3.60
Rt. Parahippocampa Gyrus↑	34	53	18	−10	−17	5.98
Rt. Middle Temporal Gyrus↑	21	18	60	8	−20	4.47
Rt. Fusiform Gyrus↑	19	20	36	−70	−17	4.78
Rt. Middle Temporal Gyrus↑	21	7	63	−31	−14	3.41
Rt. Fusiform Gyrus↑	19	10	21	−55	−8	3.36
Rt. Middle Temporal Gyrus↑	21	43	66	−16	−5	4.39
Rt and Lt. Frontal Lobe↑	10	9324	21	44	−2	7.35
Lt. Occipital Lobe↑	17	25	−12	−106	4	4.21
Rt. Occipital Lobe↑	19	54	45	−85	4	5.24
Rt. Occipital Lobe↑	19	212	27	−97	22	6.09
Lt. Inferior Parietal Lobule↑	40	12	−51	−40	25	3.85
Rt. Inferior Parietal Lobule↑	40	10	54	−28	25	3.48
Rt. Postcentral Gyrus↑	1	13	54	−28	43	3.85
Rt. Inferior Parietal Lobule↑	40	26	51	−43	55	4.83
**MCI vs. NOR**↓						
Rt. Cerebellum Posterior lobe↓	-	5	33	−76	−50	−3.91
Lt. Cerebellum Posterior lobe↓	-	20	−3	−70	−50	−4.53
Lt. Cerebellum Anterior lobe↓	-	5	−36	−40	−41	−3.69
Rt. Cerebellum Anterior lobe↓	-	31	42	−43	−32	−5.19
Rt. Cerebellum Posterior lobe↓	-	10	24	−73	−17	−4.11
Rt. Temporal lobe↓	42	7	66	−7	10	−4.56
Lt. Superior Frontal Gyrus↓	6	60	−24	−10	73	−5.28
Rt. Middle Frontal Gyrus	6	7	27	5	70	−4.01
Rt. Superior Frontal Gyrus↓	6	16	21	−10	70	−3.64
Lt. Precentral Gyrus↓	6	5	−42	−10	61	−4.06
Lt. Paracentral Lobule↓	6	52	−3	−28	52	−5.20
Rt. Postcentral Gyrus ↓	3	222	42	−28	61	−6.06
Lt. Superior Parietal Lobe↓	5	66	−30	−55	67	−5.15
Lt. Paracentral Lobule↓	3	18	−15	−28	76	−4.20
Rt. Lingual Gyrus↓	18	8	6	−79	−11	−3.88
Rt. Limbic Lobe↓	-	19	24	−10	−35	−4.16

**Figure 4:**
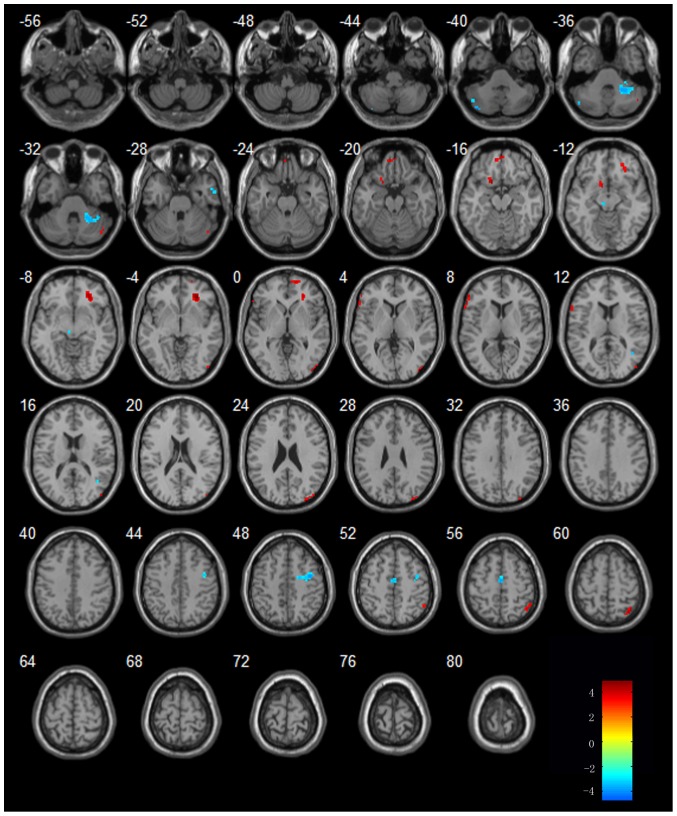
Regions showing increased or decreased activities in AD subjects in the procession of acupuncture comparing to resting state. Left in picture is left in the brain. The color scale represents t values.

In AD patients, the regions of right CPL, bilateral frontal lobe, right inferior parietal lobule (IPL), right middle occipital lobe (MOG) showed increased activities from that of resting state. Additionally, In AD patients, the regions of right superior temporal gyrus (STG), right MTG, bilateral MFG and left brain stem showed decreased activities from that of resting state. The details of these regions see table 4 and Figure 4.

**Table 4:** Regions showing increased or decreased activities in AD subjects in the procession of acupuncture comparing to resting state.

Regions	BA	Cluster	Coordinates (MNI)			T-score
		Size	x	y	z	
**AD vs. NOR**↑						
Rt. Cerebellum Posterior lobe↑	-	7	51	−67	−32	4.14
Lt. Medial Frontal Gyrus↑	11	18	0	53	−20	4.46
Lt. Inferior Frontal Gyrus↑	47	31	−12	11	−14	4.78
Rt. Middle Frontal Gyrus↑	47/11	61	27	38	−5	4.89
Rt. Superior Frontal Gyrus↑	10	12	45	−85	1	3.74
Lt. Inferior Frontal Gyrus↑	45	26	−57	29	7	4.31
Rt. Inferior Parietal lobule↑	40	36	42	−58	58	4.37
Rt. Middle Occipital Gyrus↑	19	12	21	62	−2	3.83
Rt. Middle Occipital Gyrus↑	19	20	33	−91	25	4.44
**AD vs. NOR**↓						
Lt. Cerebellum Posterior lobe↓	-	8	−39	−79	−41	−4.69
Lt. Cerebellum Posterior Lobe↓	-	11	−48	−67	−38	−3.59
Rt. Cerebellum Anterior Lobe↓	-	66	30	−49	−35	−4.10
Rt. Middle Temporal Gyrus↓	21	10	57	2	−29	−3.74
Rt. Superior Temporal Gyrus↓	22	5	42	−58	13	−3.44
Rt. Middle Frontal Gyrus↓	6	60	42	−1	46	−4.07
Lt. Medial Frontal Gyrus↓	6	23	0	−10	55	−3.93
Lt. Brainstem↓	-	5	−6	−25	−11	−3.40

### Regions showing increased or decreased activities in MCI and AD subjects in the second resting state after acupuncture comparing to resting state

**Figure 5:**
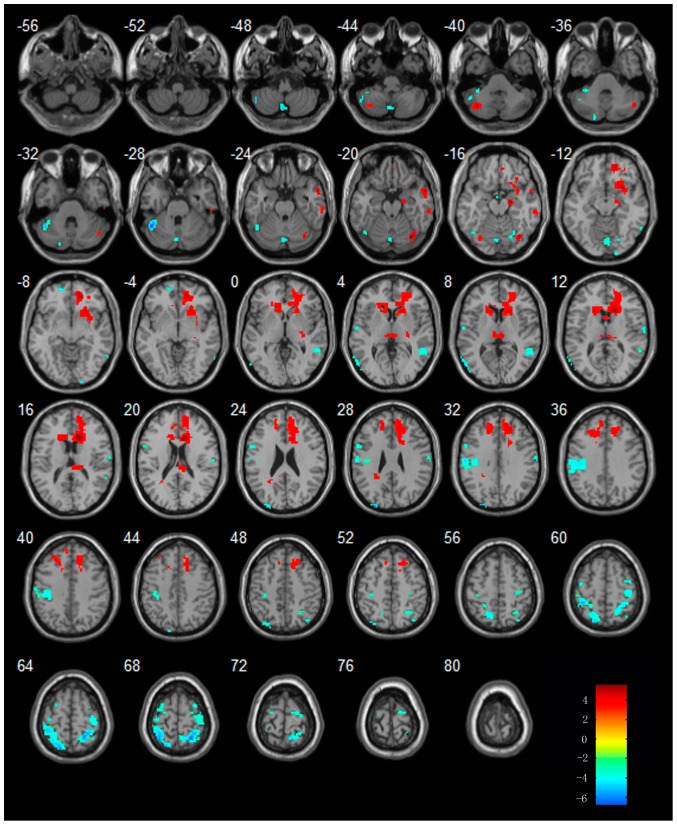
Regions showing increased or decreased activities in MCI subjects after acupuncture comparing to resting state. Left in picture is left in the brain. The color scale represents t values.

In MCI patients, the regions of bilateral CPL, temporal lobe (bilateral FG, right MTG and right PHG), frontal lobe, right lentiform nucleus, left extra nuclear and right thalamus showed increased activity in the second resting state after acupuncture comparing to resting state. Additionally, decreased activity were showed in the regions of bilateral CPL, temporal lobe (bilateral MTG, left STG, right ITG and right FG), frontal lobe (left SFG, left IFG, bilateral PrCG, right MFG), parietal lobe (bilateral PoCG, left IPL, bilateral SPL, right angular) and occipital lobe [left superior occipital lobe(SOG), left cuneus]. The details of these regions see table 5 and Figure 5.

**Table 5:** Regions showing increased or decreased activities in MCI after acupuncture comparing to resting state.

Regions	BA	Cluster	Coordinates (MNI)			T-score
		Size	x	y	z	
**MCI vs. NOR**↑						
Lt. Cerebellum Posterior Lobe↑	-	33	−36	−67	−41	4.57
Rt.Cerebellum Posterior Lobe↑		17	51	−64	−35	4.51
Rt. Fusiform Gyrus↑	19	31	36	−70	−20	5.49
Rt. Parahippocampa Gyrus↑	28	32	21	−13	−17	5.34
Rt. Middle temporal Gyrus↑	21	28	57	8	−23	4.65
Rt. Middle temporal Gyrus↑	21	33	63	−31	−17	4.09
Lt. Fusiform↑	19	7	−33	−73	−17	3.65
Rt. Frontal Lobe↑	9,8,32	1128	12	23	16	5.54
Lt. Medial Frontal gyrus↑	9	169	−18	29	34	3.95
Lt. Frontal lobe Sub-Gyral↑	-	27	−27	−43	28	3.76
Lt. Superior Frontal Gyrus↑	8	5	−9	26	49	3.37
Rt. Lentiform Nucleus↑	-	17	30	−19	1	4.03
Lt. Extra-Nuclear↑	-	165	−18	20	10	4.46
Rt. Thalamus↑	-	89	6	−22	7	4.26
Rt. Right Cerebrum sub lobar↑	-	15	0	8	13	3.38
**MCI vs. NOR**↓						
Rt.Cerebellum Posterior Lobe↓	-	7	33	−76	−50	−4.16
Lt. Cerebellum Posterior Lobe↓		24	−3	−70	−47	−5.06
Lt. Cerebellum Anterior Lobe↓		108	−42	−52	−29	−6.67
Lt. Cerebellum Posterior Lobe↓		6	−18	−85	−35	−4.06
Rt.Cerebellum Posterior Lobe↓		59	0	−70	−26	−4.69
Lt. Middle Temporal Gyrus↓	39	39	−57	−70	7	−5.17
Rt. Fusiform Gyrus↓	6	26	24	−73	−14	−4.32
Rt. Middle Temporal Gyrus↓	21	73	54	−46	4	−4.20
Lt. Superior Temporal Gyrus↓	22	9	−57	−10	7	−3.86
Rt. Inferior Temporal Gyrus↓	37	8	63	−58	−8	−3.54
Lt. Superior Frontal Gyrus↓	8	14	−15	59	−8	−5.61
Lt. Frontal lobe↓	9	10	−6	−1	−14	−4.39
Lt. Superior Frontal Gyrus↓	18	6	18	−100	−11	−3.69
Lt. Inferior Frontal Gyrus↓	44	32	−54	8	28	−4.13
Rt. Precental Gyrus↓	4	11	66	−13	31	−3.99
Rt. Precental Gyrus↓	6	124	39	−25	61	−4.54
Rt. Middle Frontal Gyrus↓	6	17	30	2	67	−4.04
Lt. Precentral Gyrus ↓	6	6	−42	−7	61	−3.72
Lt. Precentral Gyrus↓	6	13	−36	−19	67	−4.41
Lt. Superior Frontal Gyrus↓	6	30	−30	−7	70	−4.03
Rt. Postcentral Gyrus↓	43	22	66	−10	13	−4.30
Lt. Postcentral Gyrus↓	40	257	−54	−19	34	−4.93
Lt. Inferior parietal lobule↓	7	17	−36	−76	49	−4.11
Rt. Angular↓	7	9	36	−70	55	−3.83
Rt. Superior parietal lobule↓	7	19	30	−55	52	−3.87
Lt. Superior parietal lobule↓	7	276	−39	−40	61	−5.73
Rt. Postcentral Gyrus↓	7	189	24	−55	70	−6.66
Lt. Occipital superior lobe↓	7	6	−15	−85	46	−3.62
Lt. Cuneus↓	19	22	−27	−94	28	−5.82

**Figure 6:**
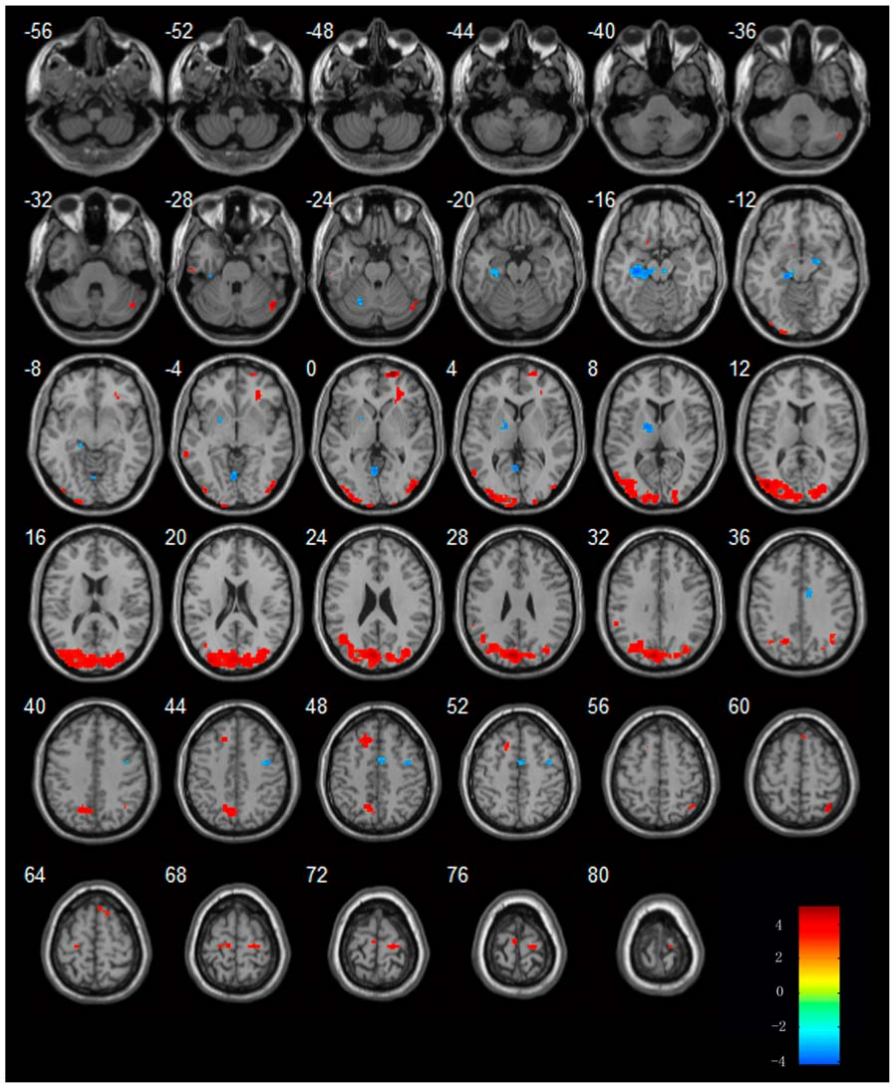
Regions showing increased or decreased activities in AD subjects after acupuncture comparing to resting state. Left in picture is left in the brain. The color scale represents t values.

In AD patients, the regions of right CPL, temporal lobe (left ITG, right MTG), frontal lobe (bilateral SFG, left IFG, right MFG and bilateral PrCG), occipital lobe(right MOG), parietal lobe(bilateral SMG, right SPL) showed increased activity in the second resting state after acupuncture comparing to resting state. Additionally, decreased activity were showed in the regions of left CPL, bilateral PHG, right MFG, left lingual gyrus, right cingulate gyrus, left lentiform nucleus and right midbrain. The details of these regions see table 6 and Figure 6.

**Table 6:** Regions showing increased or decreased activities in AD subjects after acupuncture comparing to resting state.

Regions	BA	Cluster	Coordinates (MNI)			T-score
		Size	x	y	z	
**AD vs. NOR**↑						
Rt. Cerebellum Posterior lobe↑	-	29	48	−67	−29	4.48
Lt. Inferior Temporal Gyrus↑	20	7	−60	−22	−26	4.43
Rt. Middle Temporal Gyrus↑	21	10	−66	−43	−5	3.50
Lt. Inferior Frontal Gyrus↑	47	7	−12	14	−14	3.49
Rt. Superior Frontal Gyrus↑	10	32	21	62	1	5.08
Rt. Middle Frontal Gyrus↑	47	45	30	35	−2	4.47
Lt. Superior Frontal Gyrus↑	8	52	−15	29	46	4.27
Rt. Superior Frontal Gyrus↑	6	11	3	32	61	3.42
Lt. Precentral Gyrus↑	6	11	−9	−22	67	3.34
Rt. Precentral Gyrus↑	6	30	21	−22	70	3.72
Lt. Medial Frontal Gyrus↑	6	7	−3	−16	76	3.65
Rt. Middle Occipital Gyrus↑	19	49	48	−82	−2	4.71
Lt. Middle Occipital Gyrus↑	19/18	1365	−42	−79	13	4.81
Lt. Supramarginal Gyrus↑	40	5	−54	−43	31	3.29
Rt. Supramarginal Gyrus↑	40	23	48	−52	34	3.50
Rt. Superior Parietal lobule	7	23	39	−61	61	4.46
**AD vs. NOR**↓						
Lt. Cerebellum Posterior Lobe↓	-	7	−21	−61	−23	−3.64
Rt. Parahippocampa Gyrus↓	35	12	21	−10	−14	−3.88
Lt. Parahippocampa Gyrus↓	35-	5	−33	−28	−26	−3.68
Lt. Parahippocampa Gyrus↓	35	100	−15	−25	−14	−4.10
Rt.Middle Friontal Gyrus↓	6	23	42	−1	46	−3.58
Lt. Lingual Gyrus↓	18	41	−3	−61	1	−3.95
Rt.Cingulate Gyrus↓	-	11	12	−4	37	−3.40
Rt.Cingulate Gyrus↓	-	15	6	−1	49	−3.57
Lt. Lentiform Nucleus↓	-	36	−15	−4	7	−3.95
Rt. Brainstem↓	-	5	6	−19	−14	−3.35

## Discussion

Our study used fMRI to study the regional brain activities in MCI patients, AD patients and control subject under three conditions including resting state, acupuncture and resting state after acupuncture. All subjects underwent acupuncture at four acupoints of Tai chong (Liv3) and He gu (LI4) in left and right side. We found that during the resting state, brain activities in AD and MCI patients were different from those of control subjects. During the acupuncture, AD and MCI patients showed activation in regions consistent with impaired brain function. We also found that for the resting state after acupuncture, there are several regions showing increased or decreased activities in MCI, AD subjects comparing to normal subjects. Most of regions were involved in the temporal lobe and the frontal lobe, which were closely related to the memory and cognition.

### Resting state brain activities in AD and MCI patients

Our study investigated the resting state activities in AD and MCI patients. Comparing to controls, the frontal lobe (SFG, MFG and IFG), the temporal lobe (MTG) and the lentiform nucleus showed increased activities in MCI patients. The frontal and temporal regions were considered as important components of human default-mode networks [28]–[30] and have been shown to exhibit AD- and MCI-related structural and functional abnormalities. These increases in frontal and temporal lobe could be interpreted as compensatory reallocation or recruitment of cognitive resource. This result is compatible to previous studies which showed increased temporal activation in MCI and at-risk subjects relative to healthy controls [31]–[34]. In addition, some regions such as cingulate gyrus and fusiform gyrus showed decreased activities in MCI patients comparing to controls, these changes represented the functional disruption of the above regions in the MCI patients.

Interestingly, AD patients showed different patterns of resting state activities from MCI patients. The temporal lobe and left MFG showed decreased activity in AD patients, which appeared to reflect a continuous breakdown of spontaneous brain activity during disease progression, consistent with previous studies [6], [7], [10], [12], [ and 35]. Hence, the increased frontal lobe and temporal activation has been postulated as compensatory mechanisms in MCI patients. On the other hand, the temporal lobe and left MFG exhibited decreased activities with the progression of the disease in AD patients.

### Brain activities in AD and MCI patients in the procession of acupuncture

In the current study, in order to demonstrate the value of acupuncture, we only focused on the regions which showed different activity in AD and MCI comparing to normal controls in the resting state. In MCI patients, we mainly explored changes of the left SFG, the left MFG and bilateral IFG, left MTG, the left lentiform nucleus as well as the right cingulate gyrus and left FG. In AD patients, we only focused on the left temporal lobe and left MFG.

During acupuncture, a lot of regions including the temporal lobe, the frontal lobe, the occipital lobe and the CPL showed increased activities in MCI patients comparing to the resting state. Most of these regions were related to the cognitive impairment. We noticed that the FG and cingulate gyrus were activated. On the other hand, several regions showed decreased activities in MCI patients, among these regions, we noticed that left SFG and right IFG showed decreased activities in MCI patients. To our knowledge, there were only a few previous studies using fMRI technique to explore the acupuncture effect on MCI patients. In our study we firstly found that acupuncture can modulate the brain activity in MCI patients bilaterally. That is to say, it can activate the regions which showed decreased activity in the resting state in MCI patients. It can also deactivate the regions which showed increased activity in the resting state in MCI patients.

During acupuncture, several regions showed increased or decreased activities in AD patients comparing to the resting state. However, the regions of left temporal lobe and left MFG were not be involved. We speculated that these regions probably can't be activated by the current acupoints. Future study is needed to elucidate its mechanism.

### Brain activities in AD and MCI patients in the second resting state after acupuncture

In order to examine the post-effect of the acupuncture, we also studied brain activities in AD and MCI patients in the resting state after acupuncture. A lot of regions including the temporal lobe, the frontal lobe, the limbic regions and the CPL showed increased activities in MCI patients comparing to the resting state. Most of regions were involved in the temporal lobe and the frontal lobe, which were closely related to the memory and cognition, Except for these above regions, thalamus were also activated in the procession of acupuncture in MCI patients. Zhang et al. showed that thalamus is a vital region that integrates neural activity from widespread neocortical inputs and outputs, and modulate and facilitate communication in all areas of the cerebral cortex [36]. One of our recent studies showed that disruption between the thalamus and posterior cingulate cortex (PCC) in MCI suggested the cognitive decline [37]. We noticed that the FG was activated, which showed decreased activity in the resting state. On the other hand, several regions showed decreased activity in MCI patients comparing to the resting state. Among these regions, we noticed left MTG and left IFG presented decreased activity, which showed increased activity in the resting state. This was similar with the brain activities in MCI patients in the procession of acupuncture.

After acupuncture, several regions showed increased or decreased activities in AD patients comparing to the resting state.

The activated regions include the frontal lobe, the occipital lobe, the parietal lobe and the temporal lobe. We noticed the region of left temporal lobe (ITG) was involved, which showed decreased activity in resting state. In addition, we also noticed the region of left temporal lobe (PHG) showed decreased activity after acupuncture. We speculated that the temporal lobe, as is subjected to be impaired in AD patients, was activated to compensate for the cognitive impairment.

### Conclusions

In conclusion, we investigated the effect of acupuncture in AD and MCI patients by combing fMRI and traditional acupuncture. Our fMRI study confirmed that acupuncture at Tai chong (Liv3) and He gu (LI4) can activate certain cognitive-related regions in AD and MCI patients.
